# Measurement of mouse head and neck tumors by automated analysis of CBCT images

**DOI:** 10.1038/s41598-023-39159-6

**Published:** 2023-07-25

**Authors:** Benjamin Van Court, Brooke Neupert, Diemmy Nguyen, Richard Ross, Michael W. Knitz, Sana D. Karam

**Affiliations:** Department of Radiation Oncology, University of Colorado, Anschutz Medical Campus, Aurora, USA

**Keywords:** X-ray tomography, Biomedical engineering, Software, Cancer, Cancer imaging, Cancer models, Head and neck cancer, Oral cancer

## Abstract

Animal experiments are often used to determine effects of drugs and other biological conditions on cancer progression, but poor accuracy and reproducibility of established tumor measurement methods make results unreliable. In orthotopic mouse models of head and neck cancer, tumor volumes approximated from caliper measurements are conventionally used to compare groups, but geometrical challenges make the procedure imprecise. To address this, we developed software to better measure these tumors by automated analysis of cone-beam computed tomography (CBCT) scans. This allows for analyses of tumor shape and growth dynamics that would otherwise be too inaccurate to provide biological insight. Monitoring tumor growth by calipers and imaging in parallel, we find that caliper measurements of small tumors are weakly correlated with actual tumor volume and highly susceptible to experimenter bias. The method presented provides a unique window to sources of error in a foundational aspect of preclinical head and neck cancer research and a valuable tool to mitigate them.

## Introduction

Despite advances in the use of organoids^1^ and organ-on-chip technologies^2^, in vitro systems fall short in reproducing crucial aspects of real tumors, which contain a diverse array of stromal cells, interact with a systemic immune system, and can metastasize to distant organs. Mouse models of cancer overcome these limitations and allow controlled experiments comparing groups of nearly identical animals with pharmacological and genetic manipulations to test precise biological hypotheses. They are therefore of immense value in showing that something affects tumor growth—or determining why in a context where the effect can be directly verified.

Unfortunately, the methods used to measure tumors in mice are often inaccurate, time-consuming, and susceptible to various forms of bias. The volume of an externally palpable tumor is normally approximated using the following formula (or similar) from distances measured with calipers:

1 $$V_{cal} = \frac{1}{2}d_{long} d_{short}^{2}$$

where d_long_ and d_short_ are the longer and shorter of two roughly orthogonal measurements, respectively. Details of the measurement procedure, including how tightly to squeeze the calipers, likely contribute to random errors and inconsistencies between measurers. The extent to which Eq. (1) over- or under-estimates volume is, of course, also dependent on tumor shape. This is particularly pertinent in head and neck cancer (HNC), where tumors can grow exophytically or endophytically through foramens and invade adjacent lymph nodes.

Growth of tumors that are not externally palpable, such as lung tumors^3^, or brain tumors^4^, can instead be tracked using medical imaging modalities, including computed tomography (CT)^5^, magnetic resonance imaging (MRI)^6^, and ultrasound. Although this can provide useful information, the applicability of image-based tumor measurement to serial monitoring of multiple animals is limited by lengthy image acquisition and analysis times. It has been previously reported that caliper measurement is less accurate than image-based measurement of the same tumors^7,8^. Bioluminescence imaging (BLI) is also notable in this context as a method to compare growth of appropriately labeled tumors, although it suffers from a number of technical issues^9,10^ and is not typically used to estimate tumor volume^11^.

Measurements of tumor volume by both calipers and manual image analysis, merely through the direct involvement of humans, also allow for conscious or subconscious injection of bias into the results. Despite evidence for such experimenter effects and the importance of blind data recording^12,13^, it is not a common practice in this context, and represents an important advantage of automating tumor volume quantification.

We therefore sought to develop an accurate method of tumor measurement through automated image analysis that overcomes these sources of error, while also being sufficiently cost-effective to allow for continual monitoring of tumor growth in many animals over the course of an experiment as a direct substitute for caliper measurement. To this end, we created software that processes low-dose cone beam computed tomography (CBCT) images of up to five mice with buccal tumors, isolates the individual mice, and segments the images to calculate volumes and additional information about each tumor, without requiring any user input. We present preliminary analysis of data generated by this software and caliper measurements for comparison.

Although significant obstacles still exist to widespread use of CBCT for tumor growth monitoring, we hope to demonstrate that developing technology to facilitate it has great potential utility for HNC research. It is also important to acknowledge that the methods and results described here relate to specific challenges of measuring mouse buccal tumors, which may not be relevant to tumors in other locations. Furthermore, because we do not have a gold standard measurement method against which to compare results, we cannot effectively validate the accuracy of our method as a physical measurement. These results are intended to provide a detailed description of the limitations of caliper measurement, specifically of mouse buccal tumors, and explore applications of automated CT segmentation to tumor shape and growth rate analysis, two areas we have identified as substantially benefitting from this kind of software.

## Results

### Automatic buccal tumor segmentation

**Figure 1:**
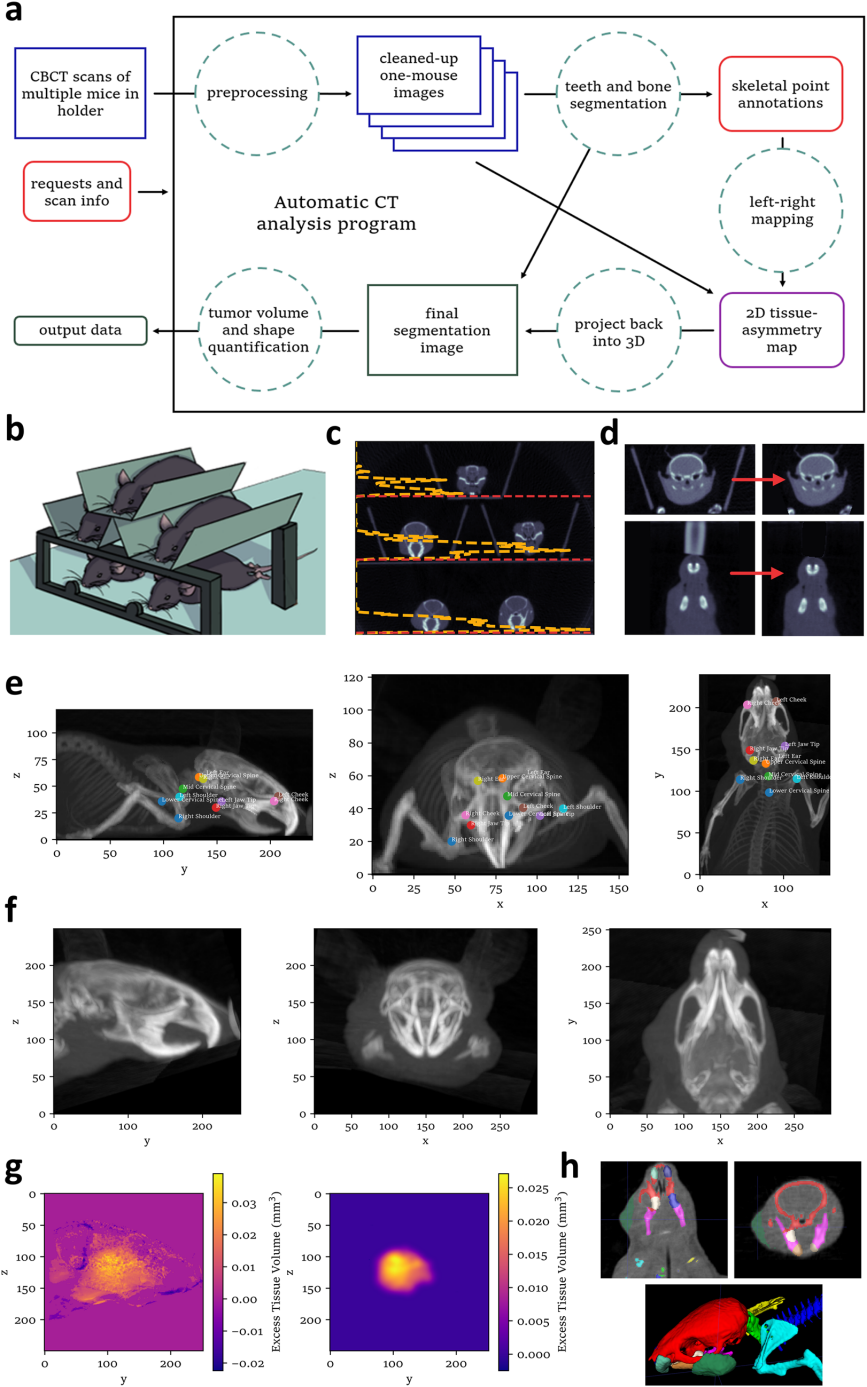
Buccal tumor growth monitoring by automatic CT analysis. (**a**) Conceptual block diagram of the automatic CT analysis program. (**b**) Illustrations of multi-mouse scanning arrangement. (**c**) Automatic splitting of multi-mouse scans. The yellow dashed line represents the number of voxels representing tooth—as isolated by thresholding—present in each horizontal plane of the scan, and the red dashed lines indicate vertical split points detected from it. (**d**) Removal of unwanted objects from scan. (**e**) Maximum intensity projections of a representative scan with automatically annotated points and arms and cranium highlighted to demonstrate bone segmentation. Figure (**e**–**g**) and Supplementary Fig. S2C–G were generated during analysis of the same scan. Axes are labeled in accordance with the coordinate system shown in Supplementary Fig. S2A. (**f**) Maximum intensity projections of the head after resampling into standard, symmetrical orientation. (**g**) (left) Initial height map of excess tissue on the right side of the head compared to the left, and (right) height map of excess tissue after 2D processing. These images are linear pseudo-color including the full range of data. (**h**) Coronal (left) and axial (right) slices of the original scan with segmented bone and tooth regions highlighted in various colors, as well as tumor in dark green, and (bottom) 3D render of segmentation as shown above.

Cone-beam computed tomography (CBCT), a form of CT imaging commonly used in dentistry and radiation therapy, is capable of quickly acquiring 3D images with high resolution in all three dimensions, which is desirable for accurate volume measurement. As a proof of concept for the use of our CBCT images for volume measurement, we scanned a collection of small aliquot tubes containing known volumes of water and used a python script to segment the image (Supplementary Fig. S1A,B). Volumes calculated from the number of voxels in each region identified as water were in very good agreement with volumes expected based on weighing the water when is was pipetted into the tubes (Supplementary Fig. S1C). In this case, image segmentation was achieved by thresholding (voxel values in water are higher than air or plastic), some processing of the binary image to correct consequences of noise and burring, and separation of the water density voxels into connected components. While similar methods are also applicable to mice, tumors are not readily distinguishable from other soft tissue in CT images, so a somewhat complicated approach was required to segment them (Fig. 1a).

Using modified stages and anesthesia setups (Fig. 1b, Supplementary Fig. S1D), we were able to acquire CBCT scans of multiple mice at a rate comparable to caliper measurement (Supplementary Fig. S1E,F), making this a viable alternative for tumor monitoring at scale. Pre-processing of the resulting scans was then required to separate individual mice (Fig. 1c) and remove non-mouse objects (Fig. 1d). The method we developed to segment each mouse exploits the symmetry of the head to identify tissue on one side that does not correspond to tissue on the other. This left–right mapping is achieved by fitting a curvilinear coordinate system that bends with the neck and jaw. In contrast to the natural coordinate system of voxel indices (Supplementary Fig. S2A), where each voxel represents the same volume of space, the curved coordinates define a grid where the volume of real space represented by each point varies. To account for this, volumes in the curved space were calculated by adding up volumes of 24 tetrahedra per voxel, arranged to count all real space exactly once (Supplementary Fig. S2B).

We used various image processing techniques, including thresholding, region growing, registration of small blocks of voxels, and filters to detect specific features, to first segment teeth (Supplementary Fig. S2C), then bone (Fig. 1e), and label various consistently identifiable points.

By matching points on the left side of the body to points on the right and interpolating between them, we then generated a curvilinear grid of points at which to resample each mouse into a symmetrical orientation (Supplementary Fig. S2D–G, Fig. 1f), which allows tissue on the left side of the head to be trivially mapped to the right and subtracted to detect tumor growth. A height map of excess tissue volume on one side was then filtered in 2D (Fig. 1g) to produce a tumor height map, which was projected back into the original 3D scan to segment the tumor (Fig. 1h).

### Repeatability of tumor volume measurements

**Figure 2:**
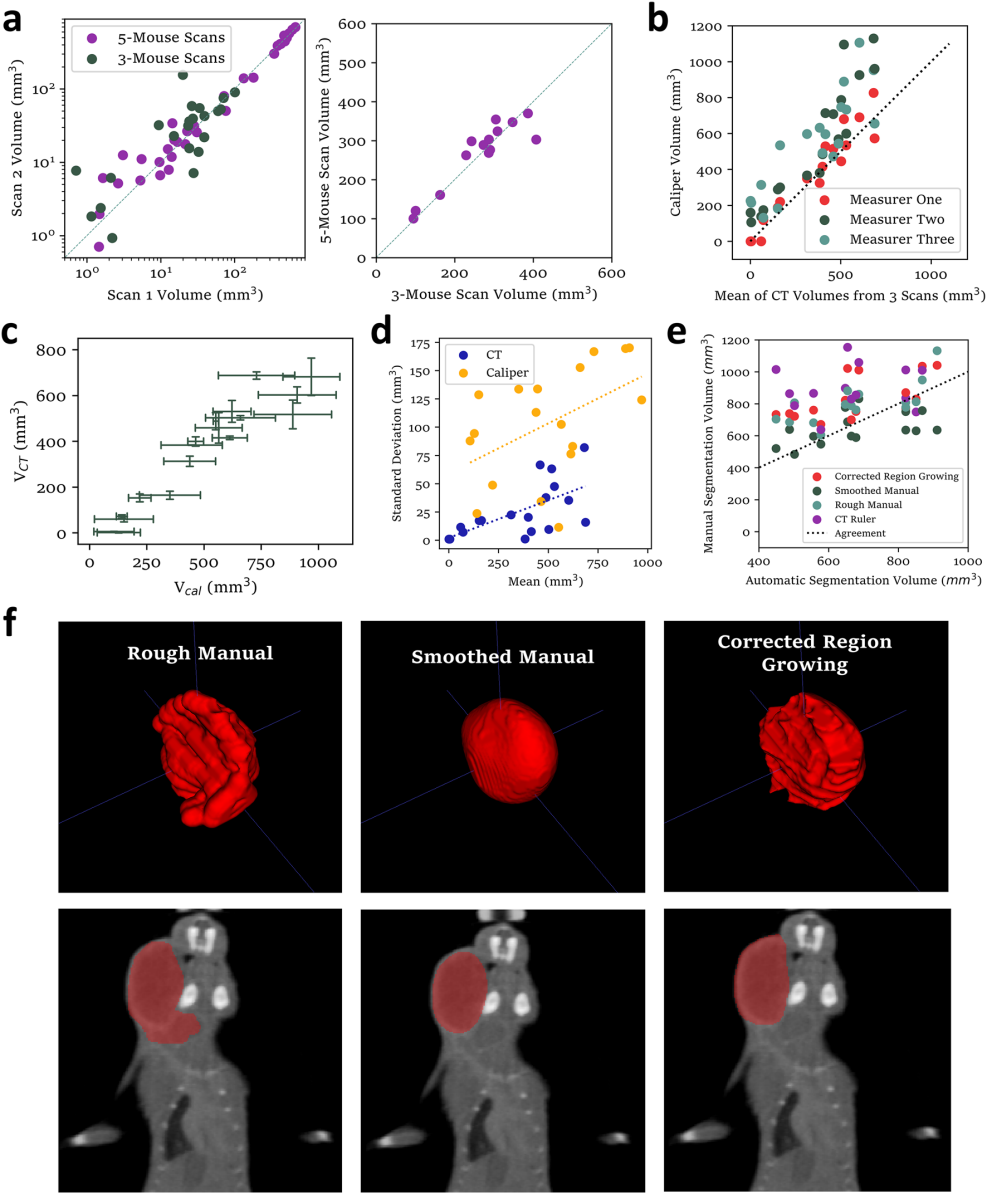
Repeatability of tumor volume measurements. (**a**) Variation between tumor volumes calculated from two scans of the same mouse on the same day. (left) for both scans using the 5-mouse scanning arrangement (Fig. 1b) with .2 mm voxels or both using the 3-mouse scanning arrangement (Supplementary Fig. S1D), and (right) from one scan of each type. (**b**) Comparison of caliper measurements taken by 3 separate measurers plotted against the average of volumes calculated from three different scans (N = 18). All other caliper measurements reported in this paper were collected by “Measurer One.” Note that panels (**c**) and (**d**) are different representations of the same data. (**c**) Variability comparison between caliper results from three measurers (horizontal bars represent their standard deviation) and 3 consecutive CT segmentation measurements (vertical bars representing their standard deviation) of the same tumors on the same day. (**d**) Comparison of standard deviations of caliper-measured volumes and CT-measured volumes for different sizes of tumor with least-squares fitted lines (dotted). (**e**) Comparison of volumes derived from manual contouring of contrast-enhance CT images by three different methods, along with CT-ruler calculated volumes (calculated using Eq. (1) from measured distances, as shown in Supplementary Fig. S3B) (N = 14). (**f**) 3D renders of three styles of manual segmentation of a representative tumor (top row) and highlighted coronal slice (bottom row) from manual segmentation of a representative contrast-enhanced CT scan.

To validate the automatic segmentation software, we scanned the same mice twice on the same day with randomized locations in each scan, to determine the extent to which calculated tumor volumes are influenced by random positioning variations. Volumes were similar between two three- or five-mouse scans, and use of lower quality five-mouse scans with 0.2 mm voxels, compared to higher quality three-mouse scans with 0.1 mm voxels, did not appear to significantly bias the calculated volumes (Fig. 2a). To compare repeatability with the caliper method, we calculated the volumes of a set of tumors from caliper measurements by three different measurers and acquired three sets of scans of the same mice on the same day. Caliper volumes varied substantially for all sizes of tumor.

One measurer tended to record lower volumes than the others, achieving good agreement with volumes from CBCT segmentation (Fig. 2b) and collected all other caliper measurements reported in this paper. Importantly, the results from caliper measurements were less similar to automatic CBCT analysis in other experiments (Supplementary Fig. S3A). Differences between measurers is a significant problem with the caliper method, resulting in obvious inconsistencies if one person does not collect all caliper measurements for a given experiment. Variability between caliper measurements of a given tumor tended to be far greater than variability between CBCT segmentation volumes from different scans, especially for small tumors (Fig. 2c). It appears that random errors in volume measurement from CT are roughly proportional to volume, whereas a significant component of the variability between caliper measurements is independent of volume (Fig. 2d).

We similarly compared results from automatic segmentation to manual segmentation. For this experiment, we acquired contrast-enhanced CBCT scans of 15 large tumors. Three different styles of manual contouring (Fig. 2e,f) were performed by two researchers, using ITK-Snap software, and measured distances approximating caliper measurement were included for additional comparisons (Supplementary Fig. S3B). We observed poor agreement between these methods (Fig. 2e). Despite visible differences between the contouring styles, it is worth noting that all of the segmentations look similar and reasonably accurate when overlayed on the scans. This suggests that for manual (or semi-automatic) image segmentation to give reproducible results would require very rigidly defined procedures. This approach is also too time-consuming to present a viable alternative to automatic segmentation for continual monitoring of many tumors.

### Comparison of tumor volumes from image segmentation to caliper measurements

**Figure 3:**
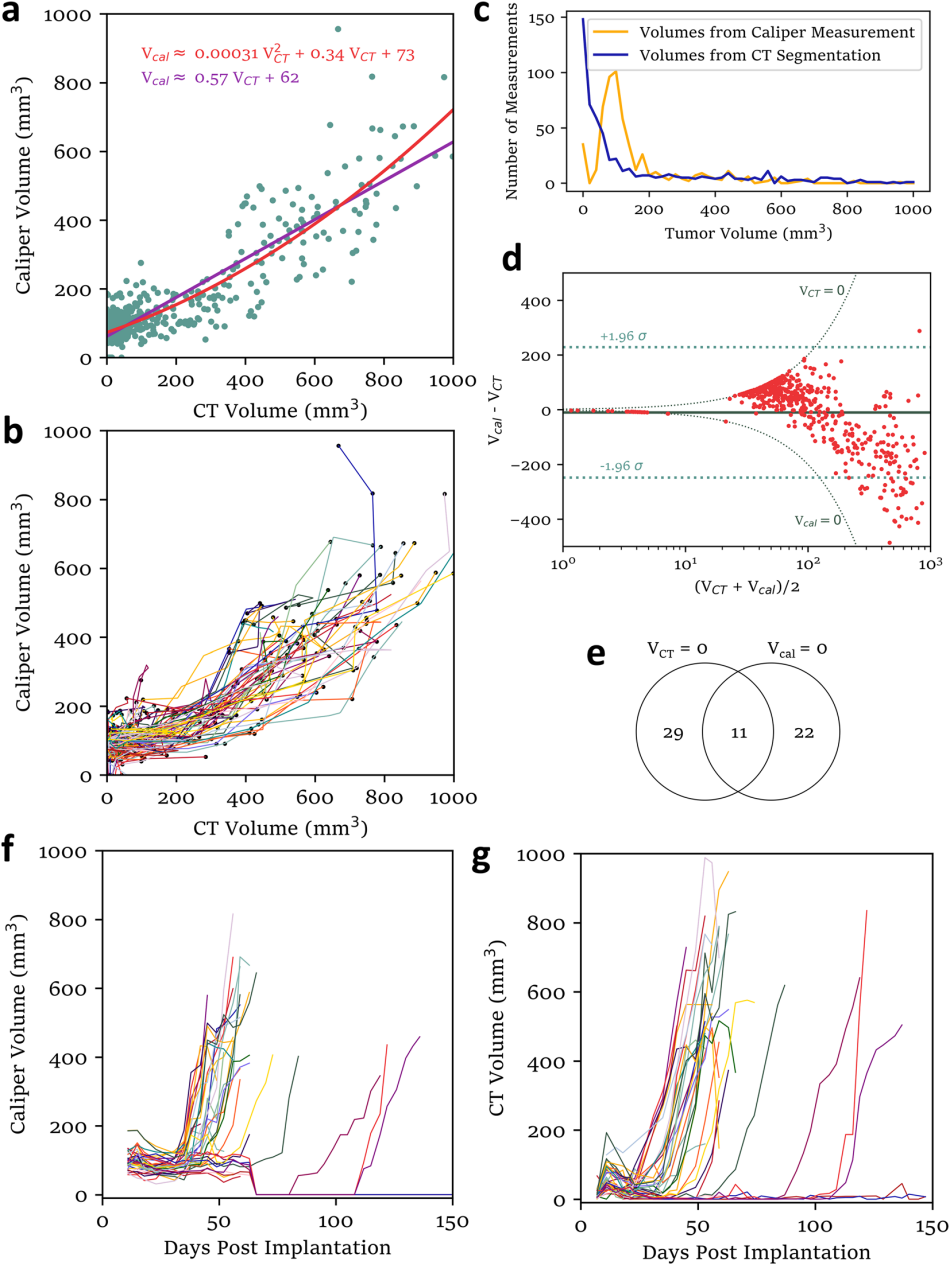
Comparison volume data from CT and caliper methods. (**a**) Matched pairs of volume measurements by caliper and CT methods of the same tumor on the same day (green) show with linear and quadratic fits. (**b**) Black dots represent matched pairs from (**a**). Connecting lines for each mouse are drawn in randomized colors and may turn at additional points, where only caliper *or* ct data is available for a particular day and the other coordinate is approximated by linear interpolation between the previous and next measurements. (**c**) Histogram of caliper and CT volumes measured. (**d**) Bland–Altman plot (with logarithmic x axis) of tumor volumes from the two methods showing volume dependence of disagreement between the two methods. (**e**) Venn diagram of tumors measured to have exactly zero volume (no tumor detected) by the two methods (from matched pairs shown in **a**,**b**). (**f**) Tumor growth curves for one experiment, as measured by calipers. Tumors were treated with 8 Gy × 3 XRT at 8, 11, and 14 days post implantation. (**g**) Growth curves for the same tumors as in panel (**f**), as measured by automatic CT segmentation. Data from N = 35 mice from one experiment are included for all panels of this figure and an additional 25 from a second experiment are included in panels (**a**–**e**).

While a linear relationship between tumor volumes from automatic CBCT segmentation and caliper measurements by the primary tumor measurer was observed when tumors were measured at a single timepoint (Fig. 2b, “Measurer One”), this was not observed when caliper measurements and CBCT scans were collected in parallel over the course of real experiments (Supplementary Fig. S3A). Two such experiments were conducted, and all matched volume pairs (CT and caliper measurements of the same tumor on the same day) are shown in Fig. 3a with linear and quadratic fits. It appears that caliper volumes less than 200 mm^3^ were nearly always greater than the corresponding CT volume and caliper volumes greater than 200 mm^3^ were nearly always less than the corresponding CT volume. A coefficient of only 0.57 for the first order term of the linear fit is particularly concerning, as it suggests that caliper measurement substantially underestimates *changes* in tumor volume.

The same data are shown in Fig. 3b, but with linearly interpolated connecting lines between points for each mouse. An important observation about these results is the large number of caliper measurements near 100 mm^3^ that correspond to CT measurements close to zero. From overlayed histograms of these volumes (Fig. 3c), we see that the dataset is primarily comprised of such measurements. A Bland–Altman plot reveals strong size-dependence of disagreement between the two methods (Fig. 3d). Both methods occasionally reported tumor volumes of exactly zero, although usually not for the same tumors (Fig. 3e).

### Effects of experimenter expectations on caliper data

**Figure 4:**
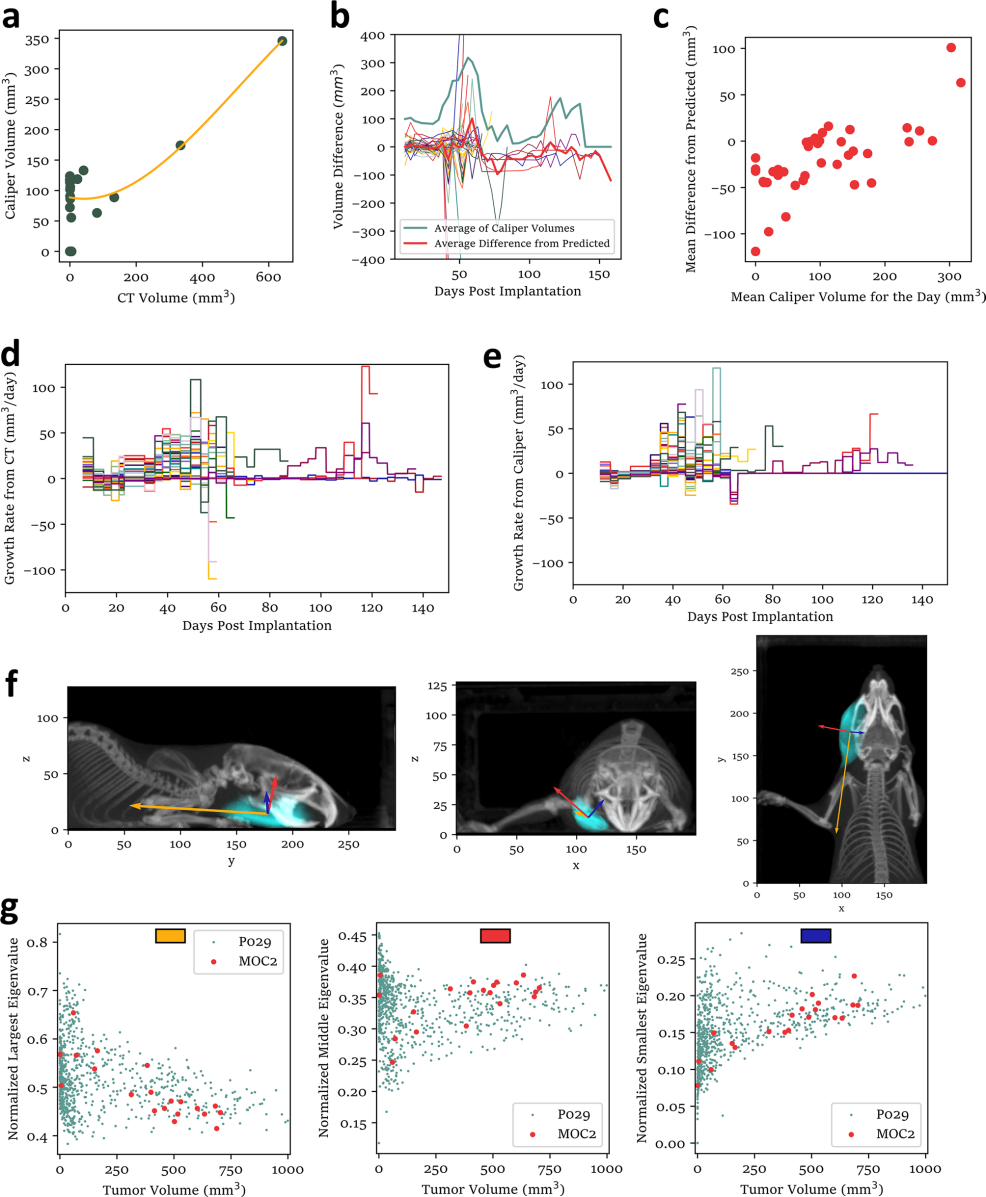
Tumor measurement by image segmentation elucidates sources of error in caliper data. (**a**) Tumor volume measurements from one representative mouse (green) with cubic fit (yellow). (**b**) Residual of caliper measurements after regressing out the effect of the actual tumor volume (thin lines), plotted with the average of these residuals from each day (red), and the average of all caliper measurements from each day (blue-green). (**c**) Scatter plot of average residual (red line from **b**) against average of caliper measurements for the day (blue-green from **b**). (**d**) Growth rates calculated by subtracting consecutive tumor volume measurements from automatic CT segmentation. These are slopes of the same growth curves shown in Fig. 3g. (**e**) Growth rates calculated by subtracting consecutive tumor volume measurements from the caliper method. These are slopes of the same growth curves shown in Fig. 3f. (**f**) Maximum intensity projections of a representative scan with tumor highlighted in cyan. Arrows are shown in the direction of the eigenvectors with lengths proportional to the square roots of the corresponding eigenvalues. Absolute scale of the arrow lengths is arbitrary and may differ slightly between panels. (**g**) Scatter plots of normalized eigenvalues of the covariance matrix of the tumor voxel coordinates (relative to centroid) for tumors from Fig. 2 (red) and Fig. 3 (blue-green). Color codes at the top of each panel indicate the corresponding arrow in (**f**). Data from N = 35 mice included in are panels (**b**–**e**). Panel (**g**) includes 20 additional mice, 18 of which were shown in Fig. 2b–d (2 were excluded by the analysis program because of problems segmenting one of the three scans).

To further investigate discrepancies between tumor volumes as measured by calipers and by automatic CT segmentation, we fitted a function to predict caliper volume from the CT volume for each mouse (Fig. 4a) and used these fits to regress out the effect of the actual tumor volume on the caliper measurement. The resulting residual measured volumes (Fig. 4b) tended to be higher on some days than others, and, in fact, correlated strongly with the average total tumor volume (Fig. 4c), suggesting a tendency to inflate reported volumes of small tumors when large tumors are present.

Notably, the measurer had direct access to measurements from previous days when recording each new set, as is common practice. This reduces the probability of reporting clearly erroneous measurements but means that the data are not truly independent between timepoints. Tumor growth curves (Fig. 3f) are likely smoothed to appear more biologically plausible through the influence of previous measurements. One undesired consequence of this can be seen in the tumor growth rates calculated by comparing consecutive volume measurements from each method (Fig. 4d,e). In this experiment, several tumors spontaneously and unexpectedly shrank. The caliper measurer noticed this but was vocally skeptical of the negative growth rates, remeasured, and ultimately recorded fewer and less dramatic reductions in tumor volume than the automatic segmentation results eventually confirmed.

### Effects of tumor shape on caliper data

We considered the tendency to record similar consecutive measurements of the same tumor as a possible contributor to the observed discrepancy between Figs. 2c and 3a (plotted together in Supplementary Fig. S3A) but chose to investigate an alternative explanation based on differences in tumor shape. The tumors measured for Fig. 2 were derived from the MOC2 cell line and mostly untreated, whereas the tumors measured for Fig. 3 were derived from the P029 cell line and treated with radiotherapy. It was therefore plausible that caliper measurements tended to overestimate MOC2 tumor volumes and underestimate P029 tumor volumes because of a difference in shape. To investigate this, we computed the eigendecomposition of the covariance matrix of the coordinates of each voxel of tumor relative to its centroid (essentially principal component analysis). A convenient geometric interpretation of this is that, for a perfectly ellipsoidal tumor, the volume would be proportional to the product of the square roots of the eigenvalues. For these tumors, the two largest eigenvalues would roughly correspond to the distances measured by calipers (Fig. 4e,f, Supplementary Fig. S3B).

To compare tumor shapes between the two experiments, we “normalized” the eigenvalues by taking the square root, then dividing by the sum of the square roots (Fig. 4f,g). MOC2 tumors tended to be more elongated than P029 tumors of similar volumes, having relatively smaller largest eigenvalues and larger middle eigenvalues. This is consistent with the higher caliper volumes reported in Fig. 2b, because the middle eigenvalue approximately corresponds to the shorter caliper distance (top panel of Supplementary Fig. S3B), which is squared in Eq. (1).

### Automatic image segmentation improves tumor growth-curve analysis

**Figure 5:**
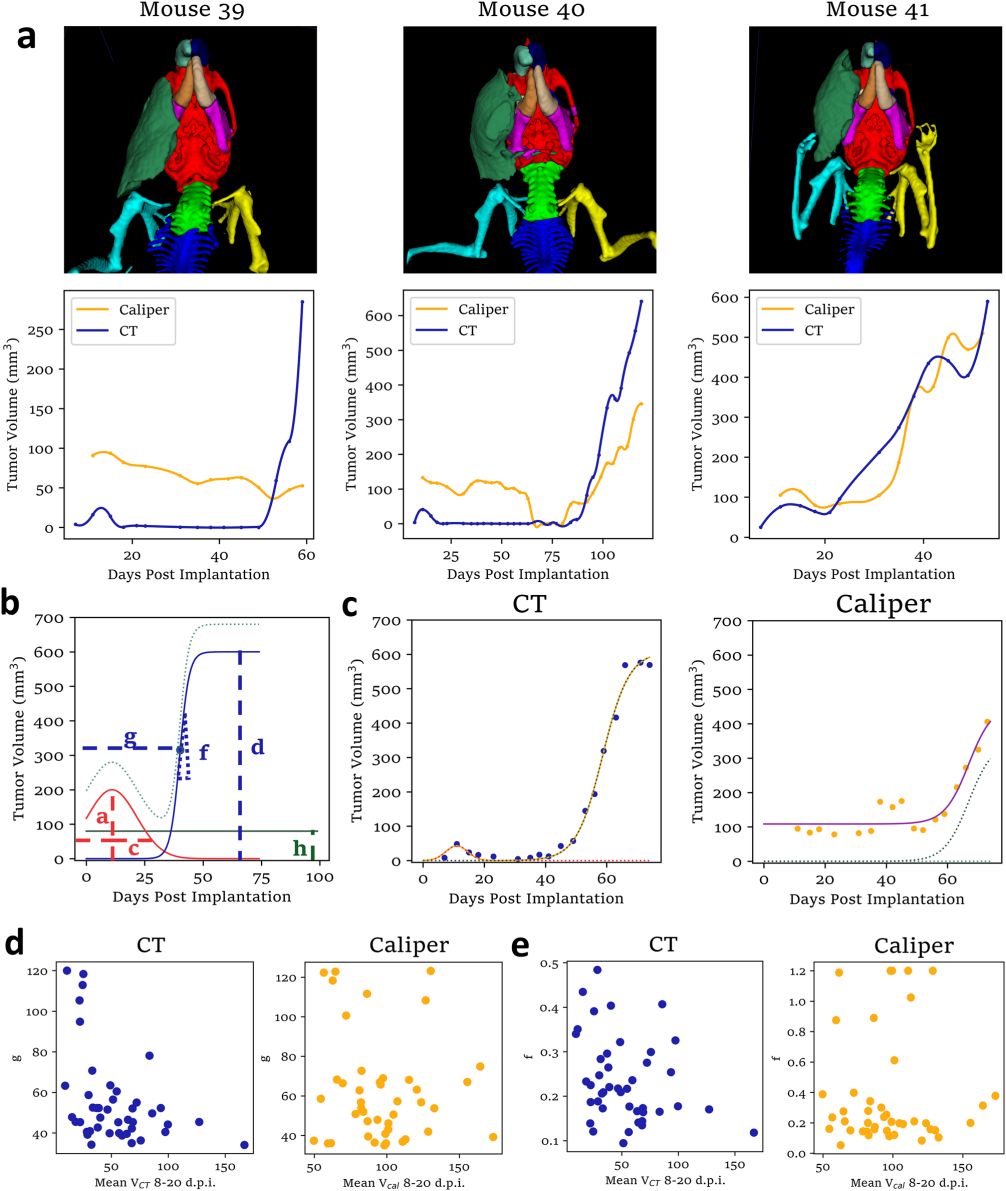
Automatic CT segmentation improves growth curve analysis. (**a**) 3D renders of 3 tumors with different shapes (top) and tumor growth curves for these tumors as measured by both CT and caliper. Connecting lines are second-order spline fits. (**b**) Model used to fit tumor growth curves for each mouse. The coefficient a represents the volume of the tumor at the time of RT, c represents the time for the tumor to shrink post-RT, d is the maximum tumor volume, f is the rate of regrowth post-RT, and g is related to the time regrowth begins post-RT. Gaussian components are shown in red, logistic components are shown in blue, and a constant is plotted in green. The light blue function is the sum of these components. (**c**) Fits to tumor growth curves from both CT (left) and caliper measurements (right) of the same representative mouse with Gaussian and logistic components (dotted). (**d**) Comparison of the mean CT and Caliper measured volumes around the time of XRT with the coefficient g shows a negative correlation in CT measurements, suggesting a relationship between average tumor volume at time of RT and time of tumor regrowth post-RT. (**e**) Comparison of the mean CT and Caliper measured volumes around the time of XRT with the coefficient f shows a negative correlation in CT measurements, suggesting a relationship between mean tumor volume at time of RT and rate of tumor regrowth post-RT. Figures (**d**) and (**e**) include N = 43 mice (all mice from two experiments, excluding tumors that never regrew).

We hypothesized that a primary benefit of more accurate tumor volume data would be more fruitful analysis of tumor growth dynamics. Growth curves for three mice are shown with in Fig. 5a with 3D renders of the corresponding tumors, showing deleterious effects of tumor shape on caliper measurement. In the first example, the tumor was very flat, causing the caliper measurer to miss it entirely (Fig. 5a, left). The next tumor is of typical shape and exemplifies the seemingly random fluctuations in caliper volume at early timepoints, followed by significantly underestimated growth rate at larger at late timepoints, observed for most mice (Fig. 5a, center). Finally, the third tumor did not respond as completely as most to radiotherapy, which is apparent from high CT volume as early as day 18, by not from caliper data until day 38 (Fig. 5a, right).

To demonstrate the utility of more accurate tumor growth curves, we developed a simple model for the growth dynamics of tumors in these experiments, where all tumors were treated with x-ray radiotherapy (XRT):

2 $$V = a\exp \left( {\frac{{ - (t - b)^{2} }}{{c^{2} }}} \right) + \frac{d}{1 + \exp ( - f(t - g))} + d + h$$

This includes a gaussian to model the initial peak around the time of XRT, a logistic curve to model the regrowth phase, and a constant term to improve fitting to the caliper dataset (Fig. 5b). This appears to capture the most important characteristics of each curve in a small number of easily interpretable parameters (Fig. 5c). We hypothesized that tumor volumes during and shortly after the time of XRT might be predictive of the parameters *f* (the fractional rate of regrowth) and *g* (the time of regrowth) characterizing the eventual regrowth phase. In CT-measured curves, we found that the average volume 8–20 days after tumor implantation negatively correlated with *f* and *g* (Fig. 5d,e). Similar trends may be present in the caliper data as well, but less clearly. Correlation statistics are given in Table 1. Interestingly, there is also a negative correlation between *f* and both the tumor’s sphericalness (Supplementary Fig. S4G) and *a* (tumor volume at the time of XRT) (Supplementary Fig. S5A), but a positive correlation between *c* (time for the tumor to shrink after XRT) and *g* (Supplementary Fig. S5B). Both *a* and *c* contribute positively to the averaged tumor volumes reported (Fig. 5d,e), yet the correlation between *c* and *g* is in the opposite direction, suggesting that the gaussian fit parameters may tease apart two separate effects: tumors that shrink slowly after XRT also take longer to enter the regrowth phase, and tumors that grow faster before XRT regrow more slowly. Similar correlations are also observed with the tumor shape metrics at early timepoints (Supplementary Figs. S4G, S5C,D). These findings require validation and explanations beyond the scope of this paper, but demonstrate how more accurate tumor measurement can reveal biologically interesting effects that would otherwise go unnoticed.

**Table 1:** Statistics for correlations involving growth curve fitting parameters.

	CBCT segmentation data				Caliper data			
	Pearson correlation		Spearman rank correlation		Pearson correlation		Spearman rank correlation	
	R	p-value	ρ	p-value	R	p-value	ρ	p-value
Mean Volume vs. *g*	− 0.4017	**0.0076**	− 0.3804	**0.0119**	− 0.1012	0.5187	0.0506	0.7473
Mean Volume vs. *f*	− 0.2991	0.0513	− 0.2945	0.0553	0.0058	0.9708	0.0017	0.9916
*a* vs. *f*	0.2954	0.0545	− 0.3958	**0.0086**	0.2406	0.1201	0.2865	0.0625
*c* vs. *g*	0.4411	**0.0031**	0.2728	0.0768	0.2192	0.1579	0.0038	0.9807

Significant are in value [bold].

## Discussion

One notable feature of the caliper growth curves shown in Fig. 3f is that several abruptly drop from about 100 mm^3^ to zero at day 60, when it was assumed that the surviving mice had been cured. A mouse with a tumor that would be measured as having a volume of 100 mm^3^ is, in fact, not necessarily distinguishable from a mouse with no tumor at all. At small volumes, the tumor is not superficial but somewhere in the cheek muscle, making caliper measurement unreliable. Furthermore, caliper measurements of small tumors tend to fluctuate together over the course of the experiment, suggesting other contextual human influence on the recorded measurements (Fig. 3f, expanded in Supplementary Fig. S3C). It is, in some sense, rational to inform the measurements with outside information, as the presence of large tumors may more accurately predict when the small tumors will start to regrow than observations of the small tumors themselves. However, it is obviously problematic from a data analysis perspective. The range of caliper volumes reported for tumors whose actual volumes were likely near zero (Fig. 4a) introduces an opportunity for the measurer to substantially bias the average volume reported for a group of mice, which might falsely confirm an incorrect hypothesis.

Interestingly, caliper volumes were not more strongly correlated with volumes from the ellipsoid approximation calculated from the eigenvalues than with the volume from voxel counting overall (Supplementary Fig. S4A), but the correlation was somewhat stronger for small tumors (Supplementary Fig. S4B), suggesting that reported caliper measurements were not related to the size of the tumor in the dimensions measured, but missing information about the thickness of the tumor makes Eq. (1) a poor approximation to the volume. This is perhaps not surprising, given that this common formula essentially uses the shorter of the two caliper measurements to approximate the thickness, which is not directly measured^14^. The assumptions of Eq. (1) have be proposed a likely explanations for inaccuracy of tumor measurement by calipers compared to CT segmentation in the literature^8^.

While the application of tumor shape analysis presented in Fig. 4f,g is somewhat mundane, it demonstrates a critical strength of tumor measurement by image segmentation: in an experiment where some tumors were treated or genetically modified in a way that affects their shape, caliper measurements might detect this as a difference in tumor volume, or simply miss the important effect. With segmented scans of each mouse, we can test new hypotheses about tumor shape and location post-hoc. For example, tumor location varies within experiments (Supplementary Fig. S4C), as does the fraction of a tumor that could fit within a sphere of the same volume (Supplementary Fig. S4D), but the MOC2 and P029 tumors show similar distributions in these metrics (Supplementary Fig. S4E,F), suggesting that the elongation of P029 tumors may not be due to increased invasion into the neck. More complicated tumor shape has been associated with poorer outcomes in HNC patients^15^, as has tumor thickness and proximity to blood vessels^16^, and automated image segmentation could significantly in generating data to determine the significance of tumor shape and location in mouse models.

In the clinic, patients’ response to therapy is often defined radiographically based on fractional changes in tumor volume. Although the widely used Response Evaluation Criteria in Solid Tumors^17^ (RECIST) are based on distances measured on medical images, as opposed to volumes from image segmentation, segmentation is thought to have superior prognostic value^18^, but at the cost of increased personnel time^19^. It is worth noting that volume is far from the only important aspect of a tumor and may not even accurately represent the number of proliferating cancer cells, as tumors vary in cellularity^20^ and my may contain necrotic regions^21^. It is however a biologically important, measurable quantity useful in comparing groups of mice with tumors that are likely to otherwise be similar. Segmented CT scans are used extensively in medical physics for Monte-Carlo simulations to determine the spatial distribution of radiation dose. Despite access to the equipment and software to perform these simulations for mice, we typically do not, because treatment planning time limits the number of mice that can be treated. Automating the segmentation could significantly improve this situation, allowing for more accurate radiation delivery tailored to the specific geometry of each tumor.

As most clinical trials fail to impact patient outcomes^22^, the translational value of preclinical models for human disease is increasingly questioned. Particularly in head and neck carcer, where immunotherapy and targeted therapy trials have uniformly failed^23^, there is a need for preclinical research to better predict the effects of drugs in human patients, which could partially be addressed by increasing the quality and quantity of data obtained from each mouse. The method of buccal tumor measurement described here contributes to this, as does detection of lung metastases, which the same scans were also used for. Software for automatic segmentation of lung tumors in CT images has been described many times in the literature^3,24–27^, and a promising future direction might be to integrate automatic measurement of lung metastases into our software to further reduce image analysis time.

Automating tumor measurement not only improves the accuracy and reliability, but reproducibility of results. Attempts to replicate preclinical cancer biology experiments are often undermined by inadequate description of methods, and find smaller effect sizes than were originally published in the vast majority of cases^28^, pointing to widespread problems in the field. Small animal CBCT imaging is available at many universities, either for image-guided radiotherapy or in stand-alone micro-CT machines. Automating the analysis of CBCT images could not only encourage more extensive use of this technology, but standardize quantification for absolute comparisons of tumor growth between experiments conducted at different institutions. This has the potential to reduce the cost in animal lives of preclinical oncology experiments, while improving their translational value to medicine.

Unfortunately, significant obstacles stand in the way of widespread use of CBCT imaging to monitor tumor growth in mice. It requires access to a suitable imaging machine, likely for many hours over the course of an experiment, which will be impossible or prohibitively expensive for many researchers, even using automated analysis of multi-mouse scans. Furthermore, our software is not perfect and only has features required for the specific imaging setup and experiments we designed it for, meaning that additional code would likely have to be written for it to be used at other institutions. While the segmentation method described here can only be used to measure tumors that grow on one side of the head or neck, it is notable that the scanning method and much of the code would also be applicable to measurement of lung and flank tumors and perhaps other locations where tumors can be readily identified in CT images. A simpler extension of the existing code might be to essentially register the image of each mouse with a different scan rather than its mirror image to eliminate the need for tumors to be confined to one side of the head.

A notable limitation of this study is the lack of a gold standard method of volume assessment to determine the absolute accuracy of the method. A seemingly reasonable solution to this problem would be to dissect out the tumor when each mouse is euthanized and measure its actual volume either by liquid displacement or weigh it to calculate volume from its expected density. Unfortunately, this did not appear to be a viable option for the experiments presented here, because the tumors extensively invaded adjacent normal tissue and by the time the mice were euthanized and complete, accurate removal of the tumor without normal tissue may not be feasible. In the absence of this kind of data we cannot report on the absolute accuracy of the method with confidence.

Another limitation of the current manuscript is the small number of tumor models examined. Different implantation sites, cell lines, and treatments may give somewhat different results. Our findings would be applicable to a broader range of preclinical oncology experiments if we had developed an automated method to measure flank tumors, used to study many types of cancer. However, it is likely that the caliper method is more accurate for measuring tumors in the flank than the buccal, because it is less likely that the tumor will be obscured by normal tissue. Notably, serious limitations of the caliper method for measuring mouse buccal tumors were obvious before any of these data were collected, simply because it is often difficult to decide where to place the calipers or if a tumor is present. This is not necessarily the case for tumors in other locations and these results should not be taken to apply to caliper measurements in general.

Our software could also likely be improved to give more accurate results with further development of various modules. For example, we did not incorporate any information about soft tissue anatomy, which might be used to make better estimates of tumor depth or more accurately locate the midline when the neck is bent. The accuracy of our tumor segmentations is also unavoidably limited by the imaging modality. Fairly low-quality CBCT scans were used to facilitate quickly imaging large numbers of mice, but high-resolution MRI or contrast agents could, in principle, be used to generate more accurate segmentations by incorporating information from soft tissue contrast.

Despite these technical challenges, our software has several clear practical applications for preclinical HNC experiments. One is to randomize groups after implantation but prior to treatment to ensure initially similar tumor volume distributions. Another is to compute tumor volume fold-changes; it could be useful to compare relative changes in tumor volume from onset of treatment, but this clearly would not be appropriate for the data shown in Fig. 3f, because the volumes from caliper measurement at the time of XRT were unrealistically high and appear to be very weakly correlated with the actual volumes of the tumors. Tumor monitoring by automated image segmentation opens the door to more sophisticate analysis of tumor growth, such as shape comparisons and fitting mathematical models, and has great potential to positively impact how preclinical HNC experiments are conducted.

## Methods

### Animal models

All methods were performed in accordance with the relevant guidelines and regulations. Animal procedures were conducted in accordance with protocols approved by the University of Colorado, Anschutz Medical Campus institutional animal care and use committee (IACUC). Some mice were euthanized due to tumor volumes exceeding 1000 mm^3^ or weight loss exceeding 5% of body weight per day over 2–3 days, or abnormal breathing due to lung metastases. Others were euthanized without reaching these endpoints because of tumor ulceration. The euthanasia method employed was carbon dioxide inhalation followed by cervical dislocation, consistent with the American Veterinary Medical Association’s guidelines for the euthanasia of animals. All experiments are reported in accordance with ARRIVE guidelines^29^.

The images analyzed for this manuscript were collected for experiments designed to explore roles of ephrinB2/EphB4 signaling in head and neck cancer. The effects of these proteins on tumor growth are, however, beyond the scope of this manuscript, so pooled data from multiple conditions are presented without identification. All analyses include all mice from the experiment or experiments presented. C57BL/6 wild-type or EfnB2^fl/fl^Tie2-Cre-ERT (genetically modified C57BL/6) mice implanted with either 100,000 MOC2^30^ or 50,000 P029^31^ cells in the right buccal. All mice were approximately 3 months old at the time of tumor implantation and female except for data from 25 males included in Figs. 3a–e and 5d,e. The MOC2 cell line was obtained from Dr. Ravindra Uppaluri (Dana-Farber Cancer Institute, Boston, MA)^32^, the P029 cell line from the Xiao-Jing Wang lab (University of Colorado, Anschutz Medical Campus). Genetic manipulations (to knock down/out EphB4) of the MOC2 and P029 cell lines were performed by the University of Colorado Cancer Center Functional Genomics Facility. MOC2 cells were transfected with PX458 control plasmid or PX458 containing gRNA targeting EPHB4 and CRISPR knockout (The same cells were used in Bhatia, et al., 2022)^32^. P029 cells were transduced with shRNA targeting murine EphB4 or non-specific shRNA. Some mice were also treated with TNYL-RAW-Fc (EphB4 inhibitor) or PCDNA3 (control) plasmids, as in Bhatia et al.^33^, except that two doses of plasmid were administered prior to tumor implantation. The procedure for implantation of buccal tumors was previously described in Oweida, et al.^14^. All tumors were treated with 8 Gy × 3 XRT at 8, 11, and 14 days post implantation, except where otherwise noted.

### Automatic buccal tumor segmentation

The program begins by locating the teeth, which were easily identified because of their high density, and defining labels and points for front, bottom, and back teeth, which can be reliably identified in nearly all scans (Supplementary Fig. S2C). Using this as a starting point, bone was then similarly segmented to identify structures such as the jaw, cranium, and shoulders (Fig. 1e). A set of line segments connecting points at the midline and corresponding points on the left and right sides of the skeleton in the original scan were then mapped to line segments of a structure that can bend at intersections to fit the position of the mouse while preserving the lengths of the line segments (Supplementary Fig. S2D). A local resampling grid to rotate and translate part of the source scan into the orientation shown in Fig. 1f is calculated for each line segment, then these are stitched together to form a single curvilinear resampling grid. Supplementary Fig. S3E shows center slices of a 3-dimensional checkerboard pattern resampled in the same way to demonstrate how the original image was warped.

Because the voxels of the resampled scan may represent slightly unequal volumes of real space, it was necessary to calculate these volumes. Supplementary Fig. S2F shows map of maximum volume represented by a voxel along three directions through the initial resampling grid. Red areas of these images show that the shoulders were compressed to span a standardized distance. The sampling grid is then adjusted by gradient descent, registering the head with its own mirror image to sub-pixel accuracy using a loss function that also penalizes curvature, but slightly expands or compresses some voxels (Supplementary Fig. S2G). To ensure that all real space was counted exactly once, the volume represented by each voxel of the resampled image was calculated from volumes of a flexible, space-filling arrangement of 24 tetrahedra per voxel (Supplementary Fig. S2B).

### Scan pre-processing

Once scans were obtained, they were processed to ensure that the measurement of the head was unabetted by other objects in the scan. For both three- and five-mouse scans, a coordinate system was established for consistent analysis (Supplementary Fig. S2A). Processing of the exported scans generated a series of single-mouse scans.

For source scans containing up to three mice in a single row in the XY plane, scan splitting begins by finding the acrylic platform by first taking the means of 100 evenly spaced columns of voxels to determine where mice were not resting on the bed. Along these columns, the “bed point” is identified as the z-value where the largest difference in voxel intensity is observed and annotated in combination with the associated x and y values. From these points, a plane was calculated and the voxels in the area below the plane were assigned the same intensity value as air, effectively removing it from the scan. Next, two redundant methods were used to horizontally separate the mice in the scan. First the mean voxel intensity of each YZ (sagittal) plane was taken to generate a one-dimensional signal which tends to have low values at the gaps between mice. Next, a threshold was applied to isolate voxels corresponding to teeth and a second one-dimensional signal was calculated by adding up the number of tooth-density voxels corresponding to each mouse. A set of splitting locations are then calculated by smoothing the first signal and finding local minima. If the number of minima identified is consistent with the expected number of mice in the scan, these become the final splitting points. If not, the second signal is similarly smoothed and its local maxima are taken to be centers of the mice to calculate splitting points by an alternative method.

For five-mouse scans, split points were first located along the z-axis for vertical splitting before horizonal splitting of the middle and bottom rows. Bed heights were estimated using the same method as the three mouse scans. However, three different height ranges were found for each of the three platforms, allowing them to be sorted into bottom, middle, and top platform points. These were used to determine each platform location. The tooth-finding for five-mouse scans also differed from that of three-mouse scans. The maximum voxel intensity of XY slices was obtained along the z-axis, finding the heights of each row of mice to split the scan in the z-axis. Along the z-axis, mice were split either just below the maximum tooth point in each row of mice, or just above the platform supporting the row of mice. Along the x-axis, the lower two rows were split in the middle of the scan, due to the consistency of volume scanning and the ample distance between the two mice in these rows (Fig. 1c).

Once the scans were split, they were processed further to eliminate unwanted objects in each scan (Fig. 1d). The sum of tissue-density voxels in each scan was then summed to determine whether a mouse was present in the split scan. If the scan was empty, it was not processed any further. The Python package Connected Components 3D was used to identify and remove unwanted objects, such as limbs of adjacent mice, which are sometimes present in the one-mouse scan after splitting. This method was also used to remove the anesthesia nosecone. One-mouse scans originating from five-mouse scans required additional processing to remove the sides of the mouse-holders. This was done by fitting a plane to points found on the plastic sides above the mouse in each scan, in a process similar to the bed-removal. The isolated scan of each mouse was then automatically adjusted based on the histogram of voxel values to ensure numerically similar values correspond to air and soft tissue before further analysis.

### Code architecture

All code was written in Python, using primarily the standard scientific computing packages NumPy, SciPy, Pandas, and Matplotlib, as well as NiBabel and Pydicom for reading and writing image files, and Connected Components 3D to analyze connected components of binary images. The overall architecture of the program is described below and in Supplementary Fig. S6.

CBCT scans were processed using a series of modules written in Python, which integrate a Microsoft Excel spreadsheet containing scan locations and information as a user interface. These modules can optionally return individual one-mouse scan, aligned scan of just the head, segmentation of structures including tumor, log of any processing errors or warnings, mouse ID numbers, tumor volumes, dates, locations of source and generated files, and debugging information.

The code was separated into 7 modules. Two provided the necessary foundation for working with CT scans. The first of these, “voxelhelp,” contained several functions designed to help with general 3D image processing and visualization. The next, “xradct” defined classes specific to one-, three-, and five-mouse scans obtained using the XRAD SmART and annotation and segmentation objects to create further ease-of-use for these types of scans.

The next two modules performed the bulk of processing for each scan to separate each mouse in the scan and calculate tumor volume. The module “scan_processing” split raw three- and five-mouse scans to produce cleaned-up one-mouse images with components of the mouse holder and anesthesia setup removed, which were used by the “head_segmentation” module to locate anatomical features and segment the image.

The final three modules were for organization and containment of the previous four. The “task_controller” module used the scan information from the input spreadsheet to split each scan and segment the new split scans. The “run” module provided a cleaner interface to run the code from. Finally, and additional module called “process_pool_controller” can act as a wrapper for the “task_controller,” allowing multithreaded execution so that many scans can be analyzed simultaneously, or provide an alternative interface to the other modules.

### CBCT imaging

The XRAD-SmART irradiator is equipped to scan a cylindrical volume up to approximately 10 cm × 10 cm. We used these capabilities to perform longitudinal monitoring of tumor growth and to obtain qualitative data on tumor size. Mice were anesthetized using 1–2% isoflurane concentration and placed in the XRAD-SmART in the prone position with a small in-machine nosecone to remain under anesthesia. For imaging buccal tumors, the front limbs were normally swept back behind the shoulders, to avoid possible issues with the image segmentation (Fig. 1b, Supplementary Fig. S1D). A brief fluoroscopy was performed in some cases to ensure correct positioning of all mice in the scannable volume, followed by a preliminary, low-resolution “scout” CBCT scan to select the volume to be reconstructed for subsequent images. CBCT scans were acquired using either a high-dose (0.1 mm voxels) or low-dose (0.2 mm voxels) scanning presets with 80 kVp or 60 kVp x-rays filtered through 0.8 mm of Beryllium and 2 mm of aluminum. Each mouse was then removed from the machine and returned to its cage to recover while the computer finished reconstructing the image. Scans were exported from the Pilot XRAD 1.18.3 software as DICOM files for analysis. For contrast-enhanced imaging (Fig. 2e,f), 200 µL of iohexol were injected by tail vein 15 min prior to imaging with 80 kVp x-rays and 0.1 mm voxel spacing All scans not used for manual tumor segmentation were acquired with no contrast agent.

To allow for simultaneous imaging of three mice at a time, we modified the in-irradiator anesthesia system by splitting the isoflurane tube into several smaller ones and attached them to a wide acrylic platform (Supplementary Fig. S1D). However, given that each cage in our vivarium can host up to five mice, we reasoned that scanning five mice at once would significantly increase efficiency for larger studies, as this allows for each cage to be scanned individually. To procure these scans, we designed a device to hold and anesthetize five mice. A 3D-printed frame holds small pieces of plastic in the shape of three half-pentagon slots for three mice. This assembly is then suspended above two more mice below additional 3D-printed objects glued to the acrylic platform. The five-mouse holder was designed to fit within the 10 cm diameter of the cylindrical scanning volume and hold all five mice at approximately the same distance from the gantry’s axis of rotation, which is important because image quality varies with this distance. Mice were first anesthetized in the induction chamber, then the lower two were positioned prone with their noses to the anesthesia tubes. The top level of the device is placed over the lower two mice, and the remaining mice are positioned in the three upper holders in the same position as the lower two. Mice were scanned using the lower resolution preset to decrease scan processing time.

### Timing of CBCT scans and caliper measurements

CBCT scans were timed using a combination of stopwatch data and timestamps associated with the beginning of each scan. Caliper measurements were timed using a stopwatch. The times associated with the setup of either measurement method were found to be similar, but varied enough that they were omitted from the total time data.

### Curve fitting

Growth curves shown in Fig. 5a were generated by fitting second order splines using *scipy.interpolate.UnivariateSpline*. To mitigate potential effects of caliper and CT data having been collected on different days, we also used these splines to resample the growth curves at 10 points 8–20 days post implantation, and averaged these values to obtain the tumor volumes reported in Fig. 5d. Fitting of Eq. (2) to volume data was performed using *scipy.linalg.lstsq()*.

### Statistical analysis

All correlation coefficients were calculated using the SciPy functions *scipy.stats.pearsonr()* and *scipy.stats.spearmanr()*. One way ANOVA tests comparing caliper volumes for all groups within each experiment at each day when caliper data were available for all mice were performed using the function scipy.stats.f_oneway(). The p-value for each test was > 0.05, except for the experiment shown in Fig. 3f,g, which had an ANOVA p-value of 0.04 and f = 2.85 at 23 days post implantation. This was confirmed using GraphPad Prism, but a Sidak follow up test comparing each pair of groups that differed in exactly one way found no statistically significant differences between individual groups.Adjusting the ANOVA p-values for comparisons on multiple days also yields no statistically significant differences.

## Supplementary Information

Supplementary Information 1.

Supplementary Information 2.

Supplementary Information 3.

Supplementary Information 4.

Supplementary Figures.

## Data Availability

Image analysis code will be available on GitHub. Tumor volume data are included in supplementary Excel files S1–S4. CBCT scans are available from the corresponding author upon request.
