# Supplementary material for: Measurement of mouse head and neck tumors by automated analysis of CBCT images
Source: Sci Rep. 2023 Jul 25;13:12033. doi: 10.1038/s41598-023-39159-6 (PMC10368694; doi:10.1038/s41598-023-39159-6)
Supplement: Supplementary file 5 — Supplementary Figures. [file 41598_2023_39159_MOESM5_ESM.pdf]

Measurement of Mouse Head and Neck Tumors by Automated Analysis of CBCT Images

Supplementary Materials

Supplementary Figure 1: Viability of CBCT for pre-clinical tumor monitoring at scale.

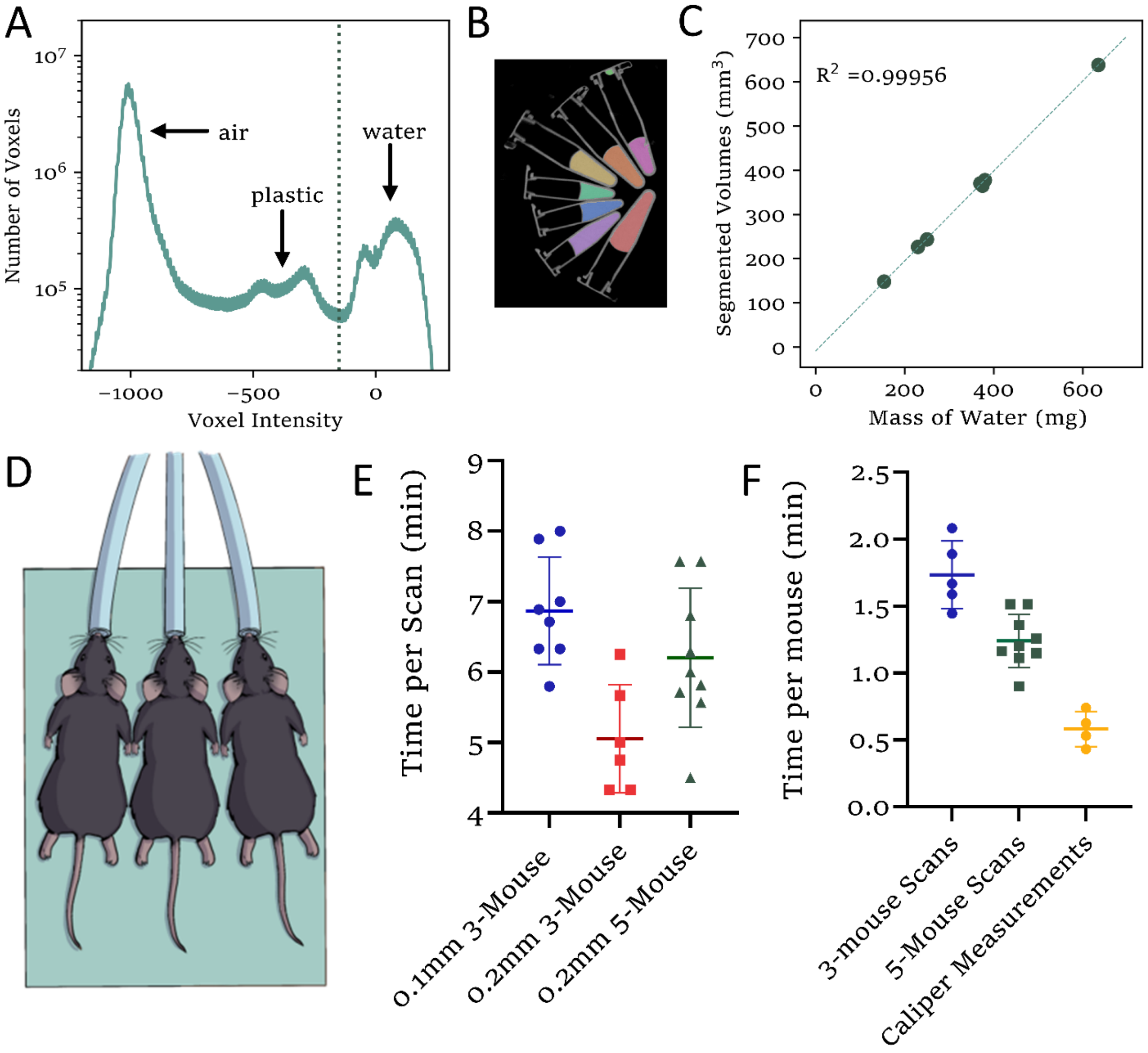

(A) Voxel intensity histogram of the scan shown in B with peaks corresponding to various materials indicated. Intensities above the dotted line are identified as water. **(B)** CBCT scan of several aliquot tubes of water with water regions highlighted in various colors. **(C)** Volume of

CBCT segmentations have good agreement with water volumes by mass. Error bars represent the standard deviation. Note that a drop of water was found at the top of one tube, so volumes of two regions of water (green and pink in B) were added for one data point. **(D)** Illustration of 3-mouse scanning setup. **(E)** Time taken (minutes) to capture different resolutions and types of scans. Low resolution 3-mouse scans are the fastest method. Error bars represent the standard deviation. **(F)** Time per mouse to obtain caliper measurements vs. 3- and 5- mouse scans. Note that the 5-mouse scans were reconstructed with larger voxels than the -mouse scans to reduce processing time.

Supplementary Figure 2: Additional images from CT analysis program.

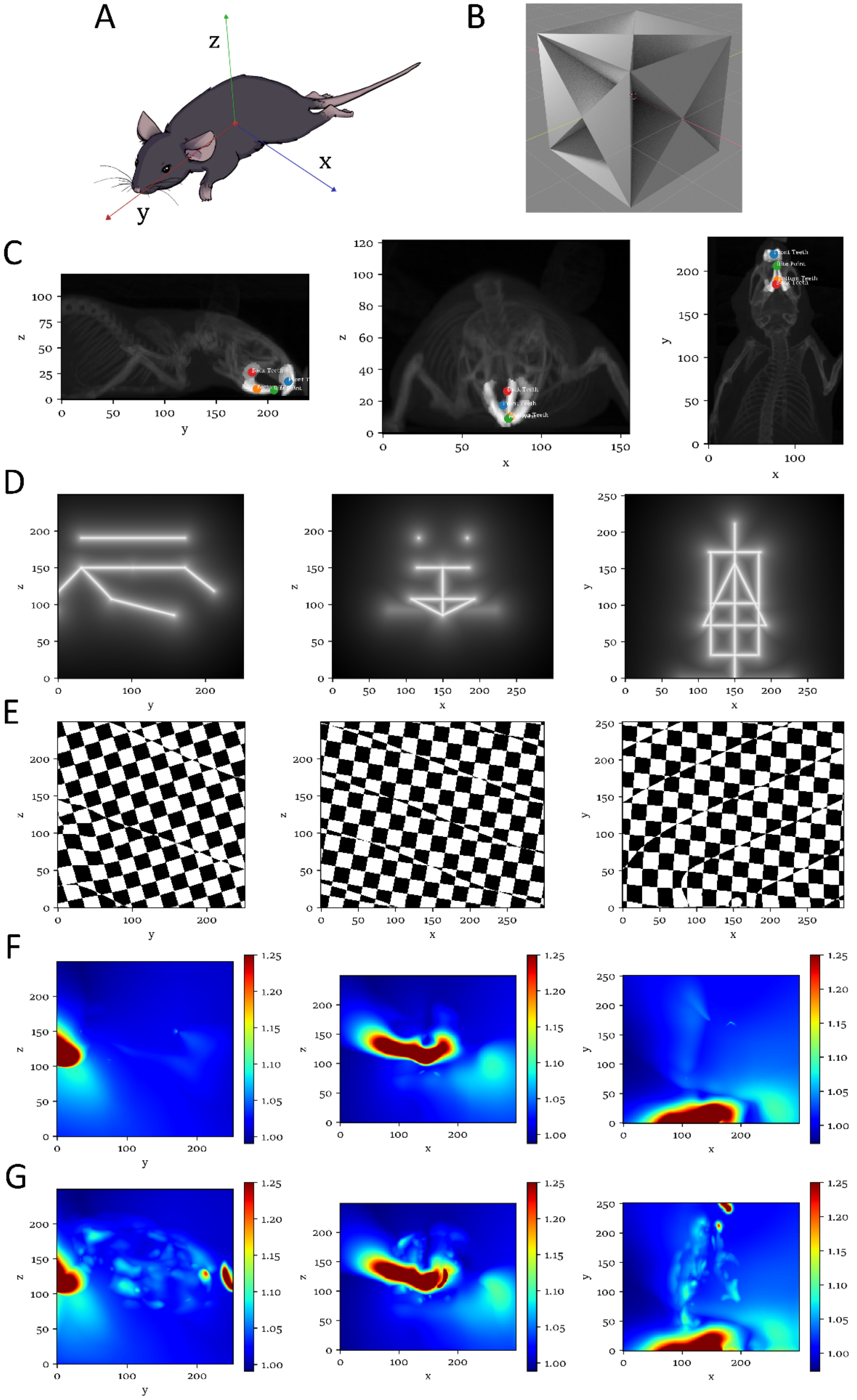

(A) Illustration of coordinate system used for axis labels. **(B)** 3D render illustrating the arrangement of 24 tetrahedra used to assign exact volumes to each voxel of the curvilinear resampling grid (see E-G). **(C)** Maximum intensity projections of a representative scan with tooth regions highlighted and generated point annotations. **(D)** "Frame" structure used to *warp* source scans into approximately the symmetrical orientation shown in Figure 1F. Ends of the line segments are defined in terms of automatically annotated points (Figure 1E, Supplementary Figure 2C) and warped into approximately this orientation for every scan (with slight variations to preserve their lengths). **(E)** Center slices of a 3D checkerboard test pattern resampled from the coordinate system of the source scan in the same manner used to generate Figure 1F. **(F)** Maximum intensity projections of the real-space volume represented by each voxel of the initial resampling grid (prior to gradient descent adjustment). The red region represents compression of the shoulders. Color scales are in nL per voxel (note that the resampled image has approximately twice the resolution of the source image in each dimension). **(G)** Maximum intensity projections of the real-space volume represented by each voxel, as shown in F, but after gradient descent correction to improve left-right mapping. Color scales are in nL per voxel. Values in F and G are clipped to the range specified by the color bar to improve visibility (LUT does not include the full range of data).

Supplementary Figure 3: Problems with caliper measurement of mouse buccal tumors.

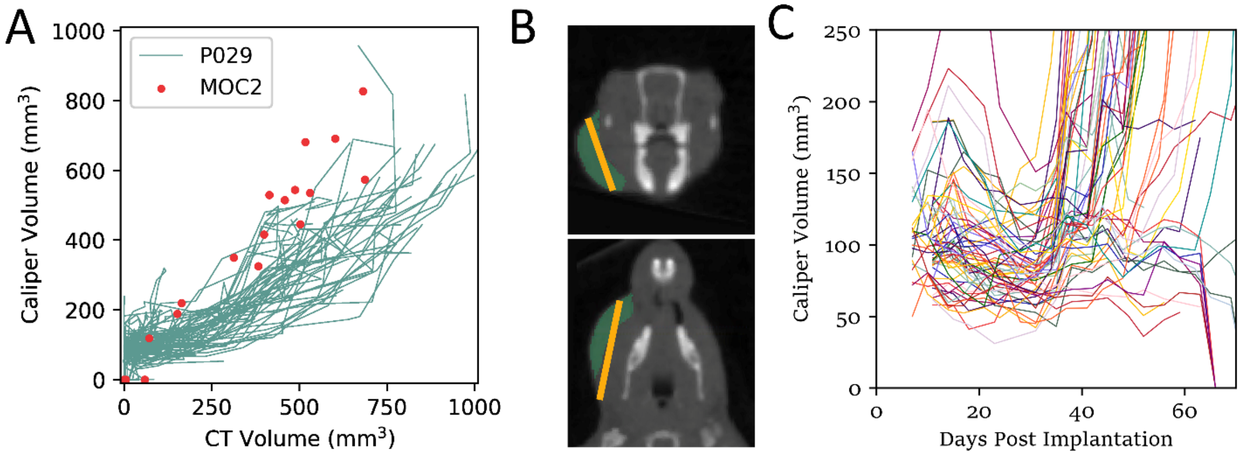

(A) Tumor volumes from caliper measurement plotted against volumes from automatic CT segmentation. Data from Figure 2B ("Measurer One") are shown in red and data from Figure 3B in blue-green. The labels "MOC2" and "P029" refer to the cancer cell lines used for these experiments. **(B)** (top) axial and (bottom) coronal slices of representative mouse with tumor highlighted in green and approximate distances measured by calipers shown in yellow. **(C)** Zoomed in view of the lower left of Figure 3F to improve visibility of fluctuations in caliper volumes reported for small tumors.

Supplementary Figure 4: Additional geometric information from tumor segmentation.

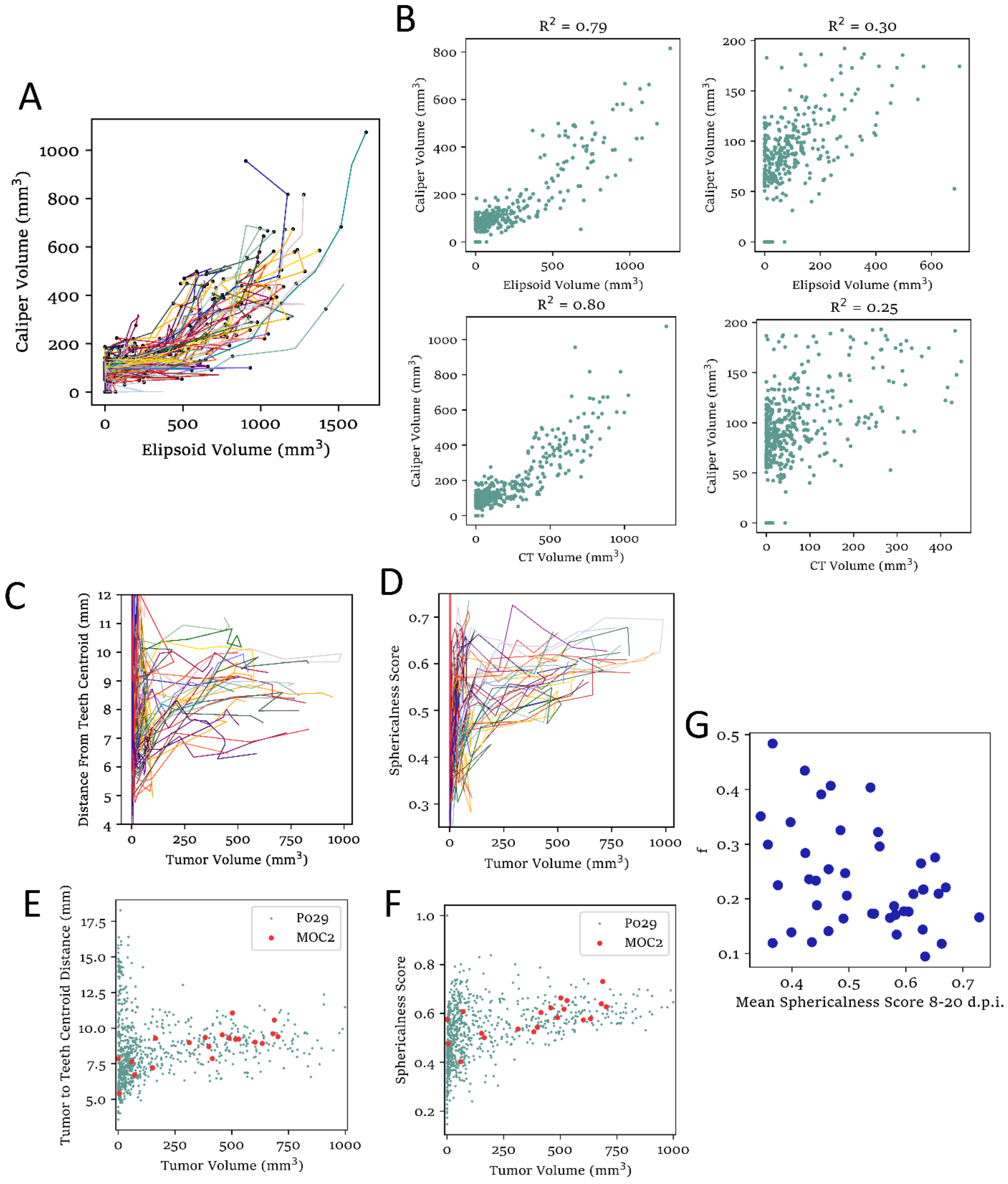

(A) Comparison of caliper volume to the volume of an ellipsoid calculated from the eigenvalues of the covariance matrix of the coordinates of the tumor voxels (as shown in Figure 4F). **(B)** (top

row) scatter plots of caliper volume against ellipsoid volume and volume from voxel counting (bottom row) for all data (left column) and only caliper measurements under 200 mm^3 (right column). **(C)** Distance between the centroid of the tumor segment and the centroid of the front and bottom teeth (a point in the tongue) for the tumors from Figure 3G. **(D)** Sphericalness score (the fraction of a tumor that could fit within a sphere of the same volume) plotted against volume for the tumors from Figure 3G. **(E)** Scatter plot of the data from C (blue-green) with additional data points from the tumors measured for Figure 2B (red). **(F)** Scatter plot of the data from D (blue-green) with additional data points from the tumors measured for Figure 2B (red). **(G)** Scatter plot of the growth curve fitted parameter f against sphericalness score around the time of XRT.

Supplementary Figure 5: Additional curve fitting results.

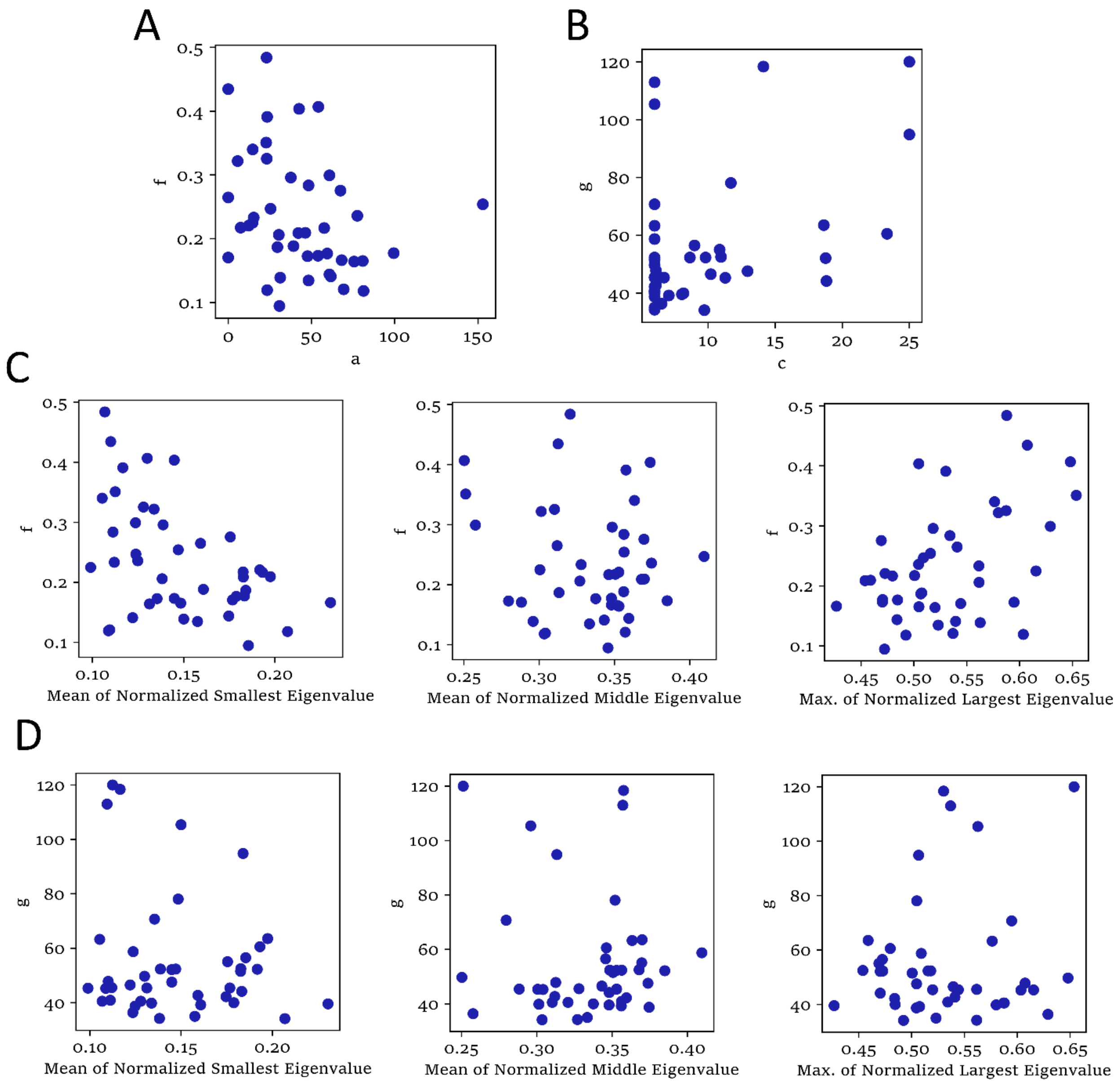

(A) Scatter plot showing some negative correlation between tumor growth curve fit parameters a (height of the gaussian) and f (slope of the logistic curve). Statistics are provided in table 1. **(B)** Scatter plot showing some positive correlation between tumor growth curve fit parameters c (width of the gaussian) and g (time delay of the logistic curve). Statistics are provided in table 1. **(C)** Scatter plots of f (slope of logistic curve from fitted model, see Figure 4F) against the mean of

each eigenvalue of the covariance matrix of the coordinates of the tumor voxels spline-averaged from 8-20 days post implantation. Statistics are given in Supplementary Table 1. **(D)** Scatter plots of g (time delay of logistic curve from fitted model, see Figure 4F) against the mean of each eigenvalue of the covariance matrix of the coordinates of the tumor voxels spline-averaged from 8-20 days post implantation. Statistics are given in Supplementary Table 1.

Supplementary Figure 6: Block diagram of software architecture.

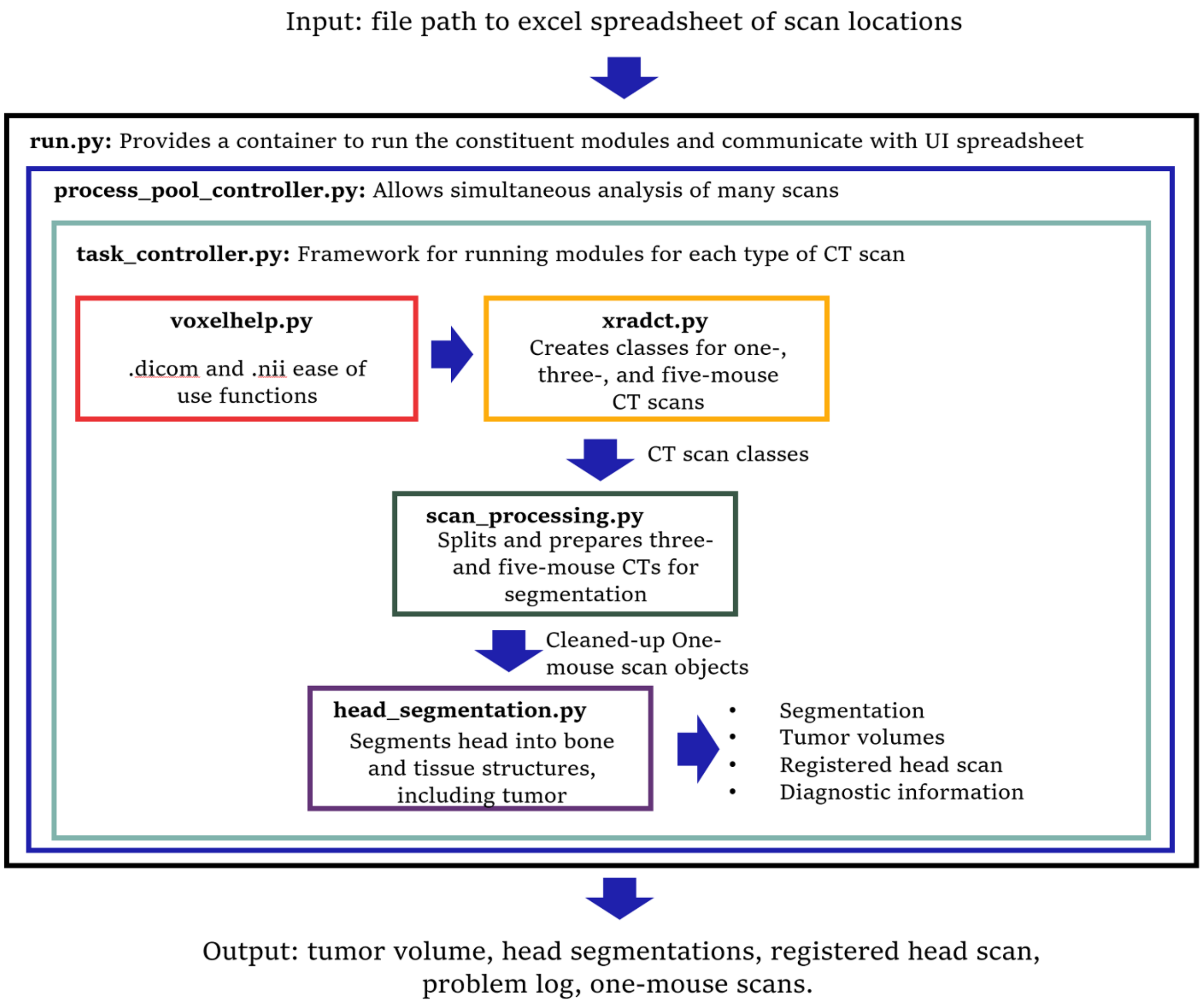

Supplementary Data Files

The following supplementary data files are also included with this manuscript:

Data 1: Three set of caliper volumes for the same tumors on the same day.

The file “Data 1.xlsx” contains the caliper data plotted in Figure 2B-D.

Data 2: Three set of CBCT segmentation volumes for the same tumors on the same day.

The file “Data 2.xlsx” contains the CT volume data plotted in Figure 2B-D.

Data 3: CBCT segmentation volumes for full experiments.

The file “Data 3.xlsx” contains the caliper volume data plotted in Figure 3-5.

Data 4: CBCT segmentation volumes for full experiments.

The file “Data 4.xlsx” contains the CT volume data plotted in Figure 3-5.
